# Differential regulation of intracellular factors mediating cell cycle, DNA repair and inflammation following exposure to silver nanoparticles in human cells

**DOI:** 10.1186/2041-9414-3-2

**Published:** 2012-02-10

**Authors:** PV AshaRani, Swaminathan Sethu, Hui Kheng Lim, Ganapathy Balaji, Suresh Valiyaveettil, M Prakash Hande

**Affiliations:** 1 Department of Chemistry, Faculty of Science, 3 Science Drive 3, National University of Singapore, 117543, Singapore; 2 Department of Physiology, Yong Loo Lin School of Medicine, 2, Medical Drive, National University of Singapore, 117597, Singapore; 3 Tembusu College, National University of Singapore, 28 College Avenue East, 138598, Singapore

**Keywords:** DNA damage, Isothermal titration calorimetry, inflammation

## Abstract

**Background:**

Investigating the cellular and molecular signatures in eukaryotic cells following exposure to nanoparticles will further our understanding on the mechanisms mediating nanoparticle induced effects. This study illustrates the molecular effects of silver nanoparticles (Ag-np) in normal human lung cells, IMR-90 and human brain cancer cells, U251 with emphasis on gene expression, induction of inflammatory mediators and the interaction of Ag-np with cytosolic proteins.

**Results:**

We report that silver nanoparticles are capable of adsorbing cytosolic proteins on their surface that may influence the function of intracellular factors. Gene and protein expression profiles of Ag-np exposed cells revealed up regulation of many DNA damage response genes such as Gadd 45 in both the cell types and ATR in cancer cells. Moreover, down regulation of genes necessary for cell cycle progression (cyclin B and cyclin E) and DNA damage response/repair (XRCC1 and 3, FEN1, RAD51C, RPA1) was observed in both the cell lines. Double strand DNA damage was observed in a dose dependant manner as evidenced in γH2AX foci assay. There was a down regulation of p53 and PCNA in treated cells. Cancer cells in particular showed a concentration dependant increase in phosphorylated p53 accompanied by the cleavage of caspase 3 and PARP. Our results demonstrate the involvement of NFκB and MAP kinase pathway in response to Ag-np exposure. Up regulation of pro-inflammatory cytokines such as interleukins (IL-8, IL-6), macrophage colony stimulating factor, macrophage inflammatory protein in fibroblasts following Ag-np exposure were also observed.

**Conclusion:**

In summary, Ag-np can modulate gene expression and protein functions in IMR-90 cells and U251 cells, leading to defective DNA repair, proliferation arrest and inflammatory response. The observed changes could also be due to its capability to adsorb cytosolic proteins on its surface.

## Background

Wide spread use of nanoparticles has increased the risk of nanoparticle induced toxic effects in the environment and in humans. The rate of exposure increased progressively over the years when engineered nanomaterials were extensively used in a variety of industries. Intentional manipulation of nanoparticle surfaces with biomolecules and chemicals to cater various applications resulted in nanomaterials with unforeseeable activity. Large scale production and improper waste disposal may elevate human exposure to them and subsequent accumulation of these nanomaterials in nature [1]. To add on to the complexity, most of the metal nanomaterials seem to be non-biodegradable and survive in nature and tissues for years [2]. Dermal or intravenous injections of nanomaterials for therapeutic applications directly expose human beings to nanomaterials whose *in vivo *activity has not been fully resolved. Recent reports in nanotoxicology suggest that the interaction and distribution patterns of these nanomaterials are diverse in different cell types [3]. In order to take the full advantage of nanotechnology, biocompatibility and the toxicology profile of nanoparticles must also be established.

Silver nanoparticles in particular, have attained more attention and are commonly used in antimicrobial agents and disinfectants from textiles, medical, pharmaceutical and electronic industries [4-6]. Rapid commercialisation of these nanoparticles was boosted by the fallacy that they are less toxic to cells and tissues than other silver salts. In reality, silver salts such as silver nitrate (AgNO_3_) which release biologically active Ag^+ ^continually in aqueous media were reported to alter electron transport chain integrity and metabolic processes [7,8]. We have previously reported that Ag-np treated cells have limited exposure to Ag^+ ^ions as Ag-np solution contained a negligible amount of free Ag^+ ^ions [9], despite the potential release of Ag^+ ^ions from Ag-np in cell culture. Data suggested that Ag-np and Ag^+ ^can induce cell death *in vitro *through a ROS-mediated apoptotic process [10,11]. Kim *et al *[10] reported that Ag^+ ^induced metal-responsive metallothionein 1b (MT1b) mRNA expression in AgNO_3 _treated cells, but not in Ag-np treated cells. Ag^+ ^also induced oxidative stress-related glutathione peroxidase 1 (GPx1) and catalase expression to a greater extent than Ag-np. However, data showed that both Ag^+ ^and Ag-np induced comparable superoxide dismutase 1 (SOD1) expression levels and similar potency in cytotoxicity. Unanimously, Ag^+ ^appeared more toxic than Ag-np suggesting that the smaller the particles get the more toxic they become when the dose is based on mass [9-11].

The proposed mechanism for Ag-np induced toxicity as shown in our previous study [9] is via mitochondrial dysfunction, reactive oxygen species release and oxidative damage. Damage to DNA can be induced through direct binding of DNA or via oxidative damage to DNA. At cellular level, silver nanoparticles can penetrate cell membrane, be deposited at various organelles, halt cell proliferation and increase apoptosis. Silver nanoparticles are also capable of damaging ecosystem as shown in affecting development of zebrafish embryos [12] and penetrating plant system [13], causing various chromosomal aberrations to the plant cells. All these reports lack essential information on the characterisation of nanoparticles employed for the study which will be relevant from a biological perspective as well. Nanoparticles purchased from different sources have different stabilising agents and have uncertain composition and purity. Toxicity of such nanoparticles varies significantly according to their material composition and proper characterisation and accumulation of data could resolve this confusion. Chung *et al *have reported that surface modification of Ag-np with phosphoryl disulphide can greatly lower their cytotoxicity [14]. Nadworny *et al *investigated the protective and anti inflammatory capacity of Ag-np in porcine skin dermatitis [15] and in human lymphocytes [16] while experiments in rats established significant inflammatory activity [17]. These discrepancies in the nanoparticle properties demands further investigation on the toxicity of Ag-np on different human cell types. We have used human fibroblasts, IMR-90 for this purpose, as they have diverse functions like wound repair and production of cytokines [18]. They are known to play a role in wound healing process [19] which makes them a suitable model for inflammation studies. Fibroblasts are essential cell types in skin (exposure through contact) and lung tissues (exposure through inhalation) [19] and hence a suitable model. Glioblastoma cells, U251 are representative of cancer cells which are derived from brain tumours. Use of cancer cells in the study helps not only in understanding the differential response between normal and cancer cells but also provides useful information on the nanoparticle mediated therapy.

Here, we report a detailed study on the potential molecular mechanisms underlying silver nanoparticle toxicity using two different cell lines. Specific pathways involved in silver nanoparticle toxicity were explored. Our results show that Ag-np could evoke inflammatory response in cells characterised by high secretion of pro-inflammatory cytokines, as detected by enzyme linked immuno-sorbent assay (ELISA). Pathway specific assays focussing on cell cycle, DNA damage, cell adhesion and extracellular matrix and cell signalling gave vital information that can be used to assess the mode of action of Ag-np in cells.

## Results

### Binding of cytosolic proteins with Ag-np

**Figure 1:**
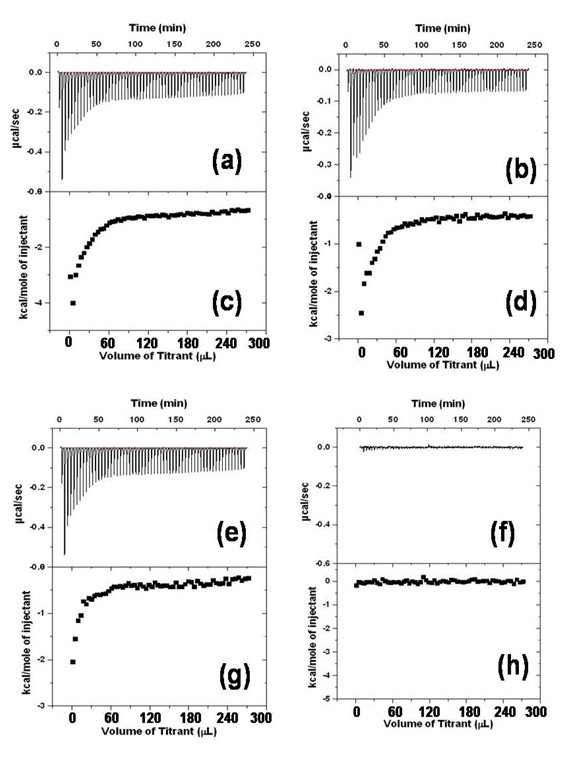
**Isothermal titration calorimetry measuring the binding of starch capped silver nanoparticles with cytosolic proteins and pure starch with cytosolic proteins**. (a) binding curves obtained by titration of cytosolic proteins to 1.8 ml of 150 μg starch capped silver nanoparticles, (b) Calorimetric traces obtained by titration of cytosolic proteins to 1.8 ml of 150 μg of starch, (c) and (d) integral data of the curves in (a) and (b) respectively plotted as a function of total volume of protein added. (e) Reaction of enthalpy of silver nanoparticles obtained after subtracting the reaction enthalpy of cytosolic proteins in 150 μg of starch capped silver nanoparticles with cytosolic proteins in 150 μg of starch without silver nanoparticles. (f) ITC of silver nanoparticles isothermal titration calorimetry of starch capped silver nanoparticles with genomic DNA. Integral curves of (e) and (f) are indicated as (g) and (h).

Isothermal titration calorimetry (ITC) emerged as a potential tool to explore the binding of DNA, proteins and amino acids with nanoparticles. A representative heat change profile for the complexation of cytosolic proteins with starch capped Ag-np and binding of cytosolic proteins with starch is depicted in Figure 1. Endothermic nature of the peaks indicates the binding between silver nanoparticles/starch with proteins. Due to the lack of exact information on cytosolic proteins such as molecular weight, concentration of individual proteins in the mixture, and number of moles of surface nanoparticles, binding isotherms were plotted against total volume of protein added. It can be seen that a monotonic decrease in the exothermic heat of binding occurs with successive injections until saturation is reached. Heat changes observed during addition of proteins may be due to binding of nanoparticle or binding of surface starch with cytosolic proteins. Control experiments were performed with pure starch in order to identify their binding with cytosolic proteins.

Interestingly, ITC curves representing the titration of cytosolic proteins with starch capped Ag-np can only be fitted into a model of two sets of binding site (Figure 1a) while the ITC titration curve representing heat change for the complexation of cytosolic proteins with starch could be fitted into a model of a single set binding sites as depicted in Figure 1b. Integral data of the curves in Figure 1a and 1b are plotted as Figure 1c and 1d as a function of total volume of protein added to Ag-np and starch, respectively. After subtraction of the binding due to starch and integration of the corresponding heat changes over time, generated a typical sigmoidal titration curve as shown in Figure 1e which strongly support the binding of Ag-np surface with cytosolic proteins. From the Figure 1f it is understood that neither starch nor Ag-np were interacting with genomic DNA. Integral data of the curves in Figure 1e and 1f are plotted as Figure 1g and 1h as a function of total volume of protein (Figure 1g) and DNA (Figure 1h), added to Ag-np.

### Effect of Ag-np on gene expression

In order to investigate the specific effects of silver nanoparticles on human cells, pathway specific gene expression profiles for cell cycle, DNA damage, cell adhesion and signalling cascades were evaluated. To assess the differential expression in a wider perspective, we have looked at these effects in both IMR 90 and U251 cells, representatives of normal cells and cancer cells respectively.

**Figure 2:**
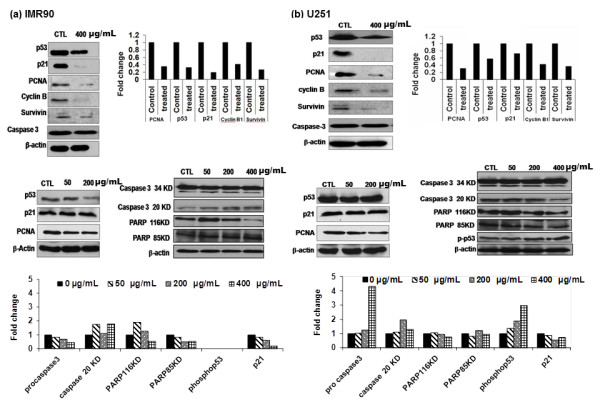
**Expression profiling of genes involved in cell cycle pathway**. Panel (a) represents western blot analysis of selected proteins showing dose dependant effect of Ag-np in IMR90 and U251 (b) cells. Values for protein levels were obtained by densitometry analysis of X-ray films using Kodak molecular imaging software and normalized against actin levels to represent accurate values for proteins which deviated from the control.

We have earlier demonstrated that Ag-np exposure affects the cell cycle process in both cells [10]. List of genes in cell cycle pathway affected by Ag-np exposure (400 μg/ml) and corresponding expression level changes are presented in Table 1. *CDC2 *was the only gene which showed a significant increase in expression in IMR90 cells, with a majority of the investigated genes down regulated (Table 1). U251 cells showed up regulation of *CDK5R2*, retinoblastoma (*Rb*) and *Bax *gene (Table 1). *Gadd 45α *in U251 cells and *Gadd 45 γ *in IMR90 cells were down regulated following exposure to Ag-np (Table 1). We show that there is a decrease in the protein expression of p53, p21, PCNA and cyclin B, which are involved in cell cycle control, following 400 μg/ml Ag-np exposure (Figure 2a, b). Protein levels for PCNA dropped by a factor of 0.35 in fibroblasts (Figure 2a) and 0.31 in cancer cells (Figure 2b). Normal cells showed a 0.32 fold drop in p53 levels whereas cancer cells exhibited a 0.58 fold decrease. There was a 0.12 fold drop in p21 levels in normal cells corresponding to 0.72 fold in cancer cells. However, a similar drop in cyclin B levels in both the cell types (~ 0.41 in normal cells and 0.42 fold in cancer cells). Low doses of Ag-np resulted in a gradual decrease in p53 and PCNA levels (Figure 2a, b) in both the cell lines. p21 showed a concentration dependant decrease in densitometry even though the protein bands appeared same intensity in control and in treated with 50 or 200 μg/mL of Ag-np (Figure 2a, b). The induction of apoptotic pathway is indicated by the phosphorylation of p53, cleavage of caspase and PARP. Ag-np exposure induced phosphorylation of p53 in a dose dependent manner in cancer cells (Figure 2b) but phosphorylation was not detected in normal cells (Figure 2a). Cleavage of Caspase and PARP was observed in both cell lines (Figure 2a, b). Density of Caspase 3 and PARP fragments were measured and presented in Figure 2a, b. Data for phospho p53 and proteins involved in apoptosis pathway (Caspase and PARP) are displayed in Figure 2a, b which showed an increase in Caspase 3 cleavage in both cell types. Over expression of Caspase 3 was observed specifically in cancer cells. Normal cells showed up regulation of PARP but no cleavage. In cancer cells an increase in Caspase and PARP cleavage was observed along with increased levels of phospho p53. We have also observed a decrease in the levels of survivin following Ag-np exposure in both the cell types.

**Table 1:** Differentially expressed genes in cell cycle pathway.

IMR 90			U251		
**Gene**	**Genbank ID**	**Fold change**	**Gene**	**Genbank ID**	**Fold change**

*CDC2*	NM_001786	24.24	*ANAPC2*	NM_013366	0.33
*CDKN3*	NM_005192	-0.67	*CDK5R2*	NM_003936	4.93
*PCNA*	NM_182649	-0.58	*RB1*	NM_000321	0.65
*SUM01*	NM_003352	-0.67	*BAX*	NM_004324	0.96
*UBE1*	NM_003334	-0.69	*CDKN3*	NM_005192	-0.87
*MCM2*	NM_004526	-0.27	*CCNE1*	NM_001238	-0.55
*BIRC5*	NM_001168	-0.22	*E2F1*	NM_005225	-0.75
*CCNH*	NM_001239	-0.27	*PCNA*	NM_182649	-0.78
*ANAPC4*	NM_013367	-0.51	*SUM01*	NM_003352	-0.37
*KPNA2*	NM_002266	-0.37	*UBE1*	NM_003334	-0.61
*CCNB1*	NM_031966	-0.91	*MCM7*	NM_005916	-0.90
*RGC32*	NM_014059	-0.61	*CCNG2*	NM_004354	-0.34
*CDC20*	NM_001255	-0.59	*CCNB1*	NM_031966	-0.75
*CCNE2*	NM_057735	-0.40	*PKMYT1*	NM_182687	-0.34
*GADD45A*	NM_001924	-0.33	*CKS1B*	NM_001826	-0.77
*RB1*	NM_000321	-0.33	*CDC16*	NM_003903	-0.21
			*MKI67*	NM_002417	-0.78
			*MAD2L2*	NM_006341	-0.56

Gene expression changes in IMR 90 and U251cells following Ag-np exposure. All values are normalized against housekeeping genes. Fold change is calculated as treated/control - 1*. *Basal levels were normalised to 0.

**Figure 3:**
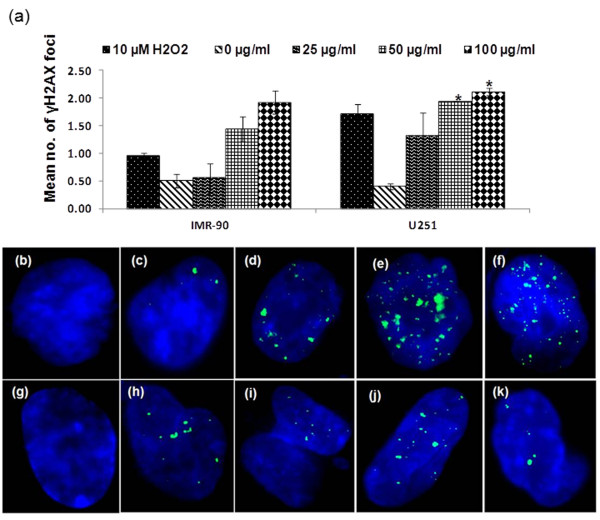
**Altered gene expression profile in Ag-np treated cells**. (a) Quantitative analysis of γH2AX foci showed more double strand DNA damage induced in U251 cells compared IMR-90 cells. Untreated U251 cells (b) showing minimal foci, (c) cells exposed to 25 μg/mL of Ag-np-3 (d) 50 μg/mL (e) 100 μg/mL and (f) 10 μM H_2_O_2_, showing multiple foci. (g) Untreated IMR90 cells shows no foci whereas, exposure with (h) 25 μg/mL of Ag-np-3 (i) 50 μg/mL (j) 100 μg/mL and (k) 10 μM H_2_O_2_, shows multiple foci.

Analysis of genes involved in DNA damage and repair pathway showed significant mRNA level changes (Table 2). U251 and IMR90 cells showed differential profiles in this pathway after Ag-np exposure. There was an up regulation of GADD45γ in IMR90 cells with rest of the observed genes were down regulated. U251 cells exhibited an increase in the major DNA damage repair pathway genes like, ATM, ATR and XPA. The other important genes in this pathway like *Abl1, Gml, Brca1, Zak, Xrcc5, Trex1, Pms1, Ccnu *and *Apex2 *were also up regulated in the cancer cells. Down regulation of genes involved in base excision repair (*Mbd4, Apex1, OGG1 and Mutyh*) and mismatch repair pathways (*Mutyh, Abl1, Pms1 and MSH2*) were observed. Double strand DNA damage was further evidenced by the appearance of γH2AX in the nuclei of Ag-np treated cells (Figure 3a). Untreated U251 cells (Figure 3b) showed generally less number of foci compared to cells exposed to 25 μg/mL of Ag-np (Figure 3c), 50 μg/mL (Figure 3d) 100 μg/mL (Figure 3e) and 10 μM H_2_O_2 _(Figure 3f). Similar pattern was observed in untreated IMR-90 cells where untreated cells showed no foci (Figure 3g) whereas, exposure with 25 μg/mL of Ag-np (Figure 3h) 50 μg/mL (Figure 3i) 100 μg/mL and (Figure 3j) 10 μM H_2_O_2 _(Figure 3k), showed multiple foci. Quantitative analysis of γH2AX foci assay confirmed that both IMR-90 cells and U251 cells showed dose dependent increase in γH2AX foci from 0 to 100 μg/mL. Higher number of foci in U251 cells suggests higher susceptibility of human cancer cells to Ag-np induced double strand DNA damage (Figure 3a).

**Table 2:** Gene expression pattern in DNA damage pathway.

IMR-90			U251		
**Gene**	**Genbank ID**	**Fold change**	**Gene**	**Genbank ID**	**Fold change**

*GADD45G*	NM_006705	6.12	*ABL1*	NM_005157	1.69
*BRCA1*	NM_007294	-0.44	*GML*	NM_002066	0.52
*ABL1*	NM_005157	-0.39	*BRCA1*	NM_007294	1.89
*GML*	NM_002066	-0.43	*ZAK*	NM_016653	2.54
*XRCC5*	NM_021141	-0.34	*ATR*	NM_001184	0.21
*MSH2*	NM_000251	-0.28	*XRCC5*	NM_021141	0.59
*RAD51C*	NM_058216	-0.74	*XPA*	NM_000380	0.91
*ERCC3*	NM_000122	-0.73	*TREX1*	NM_016381	0.51
*OGG1*	NM_002542	-0.45	*PMS1*	NM_000534	0.64
*XRCC1*	NM_006297	-0.31	*CCNU*	NM_021147	1.52
*MBD4*	NM_003925	-0.59	*APEX2*	NM_014481	0.29
*APEX1*	NM_080649	-0.67	*ATM*	NM_000051	1.98
*MUTYH*	NM_012222	-0.49	*TP53*	NM_000546	-0.28
*PMS1*	NM_000534	-0.64	*FEN1*	NM_004111	-0.51
*SUMO1*	NM_003352	-0.69	*RAD51C*	NM_058216	-0.24
*ATM*	NM_000051	-0.50	*PCNA*	NM_182649	-0.46
*CCNH*	NM_001239	-0.77	*RPA1*	NM_002945	-0.60
*IGHMBP2*	NM_002180	-0.50			
*LIG3*	NM_002311	-0.48			
*APEX2*	NM_014481	-0.29			
*PCNA*	NM_182649	-0.69			
*RPA1*	NM_002945	-0.53			

Regulation of DNA damage response genes in silver nanoparticle treated IMR 90 and U251cells. Fold change is calculated as indicated in table 1.

Array analyses of cell adhesion and extracellular matrix showed differential regulation of gene expression. Genes up regulated and down regulated for the same are listed in Table 3. *Scge *(sarcoglycan epsilon) was down regulated in both cell lines. *ITGA2 *and *Ctnna1 *were up regulated in normal cells treated with Ag-np. Additionally up regulation of integrin was observed in both cells. Transforming growth factor gene (*Tgfbi*), *Spp1 *and *ITGA5 *were up regulated in cancer cells. Matrix metallo peptidases like *MMP3 *and *MMP2 *were slightly upregulated in normal and cancer cells respectively.

**Table 3:** Gene expression changes in cell adhesion and extracellular matrix pathway.

IMR-90			U251		
**Gene**	**Genbank ID**	**Fold change**	**Gene**	**Genbank ID**	**Fold change**

*ITGA2*	NM_002203	2.32	*ITGA5*	NM_002205	1.55
*CTNNA1*	NM_001903	1.01	*SPP1*	NM_000582	1.28
*MMP3*	NM_002422	0.40	*LAMA4*	NM_002290	0.24
*ITGA11*	NM_012211	-0.83	*ADAMTS1*	NM_006988	0.26
*SGCE*	NM_003919	-0.49	*TGFBI*	NM_000358	1.44
*COL6A1*	NM_001848	-0.27	*SGCE*	NM_003919	-0.30
*LAMA4*	NM_002290	-0.27	*MMP2*	NM_004530	0.29
*ADAMTS1*	NM_006988	-0.21			

Differential expression of genes in IMR-90 and U251 cells are indicated in the table. All values are normalised against housekeeping genes. Fold change is calculated as mentioned in table 1.

**Figure 4:**
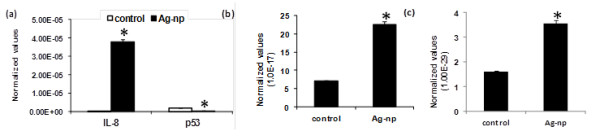
**mRNA profile as measured by RT-PCR**. (a) Elevated levels of IL-8 in IMR90 cells. (b) Increased mRNA levels corresponding to MAPK1 in normal cells. (c) NFκB mRNA levels in cancer (U251) cells. Data is represented as mean ± SE from three independent experiments. Statistical significance was determined between untreated and treated samples using Student's t test. *p ≤ 0.05.

Key molecules regulating signalling cascades showed up regulation following Ag-np exposure. *Jun, CSF2 *and *IL-8 *showed increased expression in IMR90 cells, whereas, *NFκB1, NFκBIA, FN1 *(fibronectin 1)*, GADD45A, BIRC2, TMEPAI *(prostate androgen induced RNA) *and CEBPB *(CCAAT/enhancer binding protein) were up regulated in cancer cells (Table 4). Gene data corresponding to cell signalling are represented in Table 4. Major signalling pathways involved in Ag-np mediated molecular response is indicated in Table 5. The array results were further validated by RT-PCR, where an up regulation of IL-8 mRNA levels in fibroblasts as well as down regulation of p53 (Figure 4a) was observed. MAPK1 mRNA levels were found to be higher in normal cells (Figure 4b). Exposure to nanoparticles to cancer cells showed up regulation of NFκB (Figure 4c).

**Table 4:** Genes differentially expressed in signal transduction pathway.

IMR-90			U251		
**Gene**	**Genbank ID**	**Fold change**	**Gene**	**Genbank ID**	**Fold change**

*JUN*	NM_002228	3.05	*FN1*	NM_002026	6.76
*IL8*	NM_000584	21.16	*JUN*	NM_002228	0.35
*CSF2*	NM_000758	2.82	*NFKB1*	NM_003998	26.44
*TFRC*	NM_003234	0.64	*NFKBIA*	NM_020529	1.49
*BMP2*	NM_001200	0.55	*BIRC2*	NM_001166	3.09
*BIRC2*	NM_001166	-0.22	*GADD45A*	NM_001924	17.24
*IGFBP3*	NM_000598	-0.90	*TMEPAI*	NM_020182	10.93
*IRF1*	NM_002198	-0.30	*TFRC*	NM_003234	1.55
*HK2*	NM_000189	-0.21	*CEBPB*	NM_005194	10.96
			*CXCL9*	NM_002416	- 0.86
			*IRF1*	NM_002198	-0.21

Fold change is calculated as mentioned in table 1.

**Table 5:** Summary of major up regulated genes and corresponding pathways involved in Ag-np exposure

Gene (IMR-90)	Pathways	Genes (U251)	Pathways
Jun oncogene (*JUN*)	a) Mitogenic Pathway: b) Wnt Pathway c) PI3 Kinase/AKT Pathway d) Calcium and Protein Kinase C Pathway	Jun oncogene (*JUN*)	a) Mitogenic Pathway: b) Wnt Pathway c) PI3 Kinase/AKT Pathway d) Calcium and Protein Kinase C Pathway

Bone morphogenetic protein 2 (*BMP2*)	Hedgehog Pathway	Fibronectin 1 (*FN1*)	PI3 Kinase/AKT Pathway

Interleukin 8 (*IL8*)	NFκB Pathway	Nuclear factor of kappa light polypeptide gene enhancer in B-cells 1 (p105) (*NFκB1*)	NFκB Pathway:

Colony stimulating factor 2 (granulocyte-macrophage, *CSF2*)	a) Calcium and Protein Kinase C Pathway b) LDL Pathway	Nuclear factor of kappa light polypeptide gene enhancer in B-cells inhibitor, alpha(*NFκBIA*)	NFκB Pathway

Transferrin receptor (p90, CD71), (*TFRC*)	Calcium and Protein Kinase C Pathway	Transferrin receptor (p90, CD71), (*TFRC*)	Calcium and Protein Kinase C Pathway
		
		Growth arrest and DNA-damage-inducible, alpha (*GADD45A*)	p53 Pathway
		
		CCAAT/enhancer binding protein (C/EBP), beta (CEBPB)	Insulin Pathway

### Inflammatory response in nanoparticle mediated cells

The correlation between inflammation and genotoxicity has been reported earlier [20,21]. Excessive and persistent formation of reactive oxygen species from inflammatory cells (neutrophils, macrophages) during particle-elicited inflammation will generate genetic damage resulting from the oxidative DNA attack [22]. Conversely, DNA damage is also capable of inducing the release of inflammatory cytokines [23,24]. Therefore, it is essential to investigate the inflammatory properties of Ag-np which can contribute to more thorough understanding on genotoxicity induced by Ag-np.

**Figure 5:**
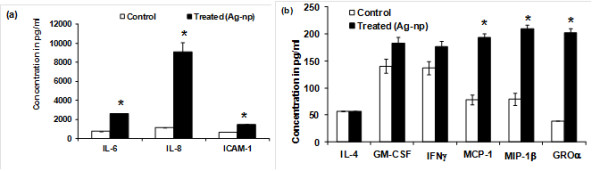
**Silver nanoparticles induced cytokines and chemokines production in normal human lung fibroblasts (IMR90)**. The graphs indicate the production of cytokines and chemokines in pg/ml (mean ± SD) by normal human fibroblasts after incubating with 400 μg/mL of silver nanoparticles for a period of 48 hours. The cytokine levels were measured in untreated cells (Control) and in cells treated with silver nanoparticles (treated (Ag-np)). Statistical significance was determined between untreated and treated samples using Student's t test. *p value ≤ 0.05.

Our study to investigate the innate ability of Ag-np to induce cytokine and chemokines production revealed that Ag-np triggered production of certain cytokines and interleukins (IL) in normal resting human fibroblasts. Of the 17 cytokines tested, only a few cytokines exhibited detectable levels in the supernatant of both Ag-np treated and untreated cells (Figure 5). There was significant increase in the production of IL-8, IL-6, ICAM-1, MCP-1, MIP-1β and GROα in those cells treated with silver nanoparticles compared to that of untreated control cells (Figure 5). There was increase in the production of GM-CSF and IFNγ in the silver nanoparticles treated cells. IL1β, IL-2, IL-7, IL-16, IL-17, TNFα, MIP1α and RANTES levels were not detected in the culture supernatants of both treated and untreated cells.

## Discussion

### Interaction of Ag-np with cytosolic proteins

Nanoparticles have a greater tendency to bind with proteins forming a protein corona [25] and mimic nutrients which facilitate easy receptor mediated uptake. Nanoparticles can also adsorb agonists or antagonists and interfere with various pathways and truncate the biomolecules [25]. Here, we show that cytosolic proteins are adsorbed on to silver nanoparticle surface forming a protein-starch coating which could control cellular responses. This observation may be useful in the understanding of intracellular behaviour of Ag-np. The surface adsorption of protein limit the availability for cellular functions, but stabilise the proteins thereby inhibiting protein degradation in proteasomes [25]. This process can alter normal cell cycle progression where timely degradation of proteins is necessary for smooth transition in to cell cycle stages. Also binding to nanoparticle surface can interfere with native conformation of the proteins leading to loss of function [25]. Another outcome of nanoparticle binding is increase in activity of proteins as a result of immobilisation at surfaces [25]. It is conceivable that the affinity and binding of Ag-np with proteins vary between cell types that have tissue specific proteins which explain the altered signalling pathways operating in different cell types after nanoparticle exposure (MAPK in fibroblasts and NFκb in cancer cells). These results set hurdles on cellular response prediction, as the identity of protein adsorbed to the nanoparticle remains unclear and the fact that the residency period and choice of protein adsorbed on to these nanoparticles varies with cell and tissue types. It is possible that Ag-np tends to have different effects on different cell types. We had reported the nuclear deposition of Ag-np in zebrafish embryos and human cells (U251) where Ag-np was seen in the nucleus of cells [12,26]. Considering the property of the nanoparticle surface which facilitates surface adsorption of proteins, we speculate that nanoparticles label cytosolic proteins which require nuclear localisation and are co-transported with them. Absence of direct interaction with genomic DNA further supports this notion. This observation has implications in nanotechnology to develop tools for nuclear targeting for cancer therapy and transfection.

### Effect on gene expression profiles

Our earlier functional studies [26] and current gene expression data showed that Ag-np exposure induced cell cycle arrest at S/G2/M phase of cell cycle. This observation can be due to the changes in the expression levels of genes regulating the cell cycle process. We have observed a decrease in the expression of p53, p21, PCNA, cyclin E and cyclin B. However the cell cycle arrest seems to be independent of p21 as there was no inhibition of cdk through p21 induced G1 arrest. Gadd 45 group of proteins are over expressed in cells upon genotoxic stress [27] was also found to be significantly elevated in Ag-np treated cells (Gadd 45α in cancer cells and Gadd 45γ in fibroblasts). Gadd 45 regulates cell cycle by repressing the localization of Cyclin B1 to the nucleus. This is known to initiate G2/M arrest in target cells [28]. Cdc 2, a binding partner of cyclin B proteins was down regulated in both cell types after Ag-np exposure. Increased Cdc 2 levels may be due to the accumulation of cells in G_2 _phase due to the decrease level of cyclin B seen earlier. *Rb *and *E2F *form a repressive complex in S phase, which upon phosphorylation by *cyclin E *releases *E2F *[29]. *E2F *being the transcription factor for S phase genes blocks cell transition through S phase [29]. We have seen that in cancer cells following Ag-np exposure there was an up regulation in the expression of retinoblastoma (*Rb*) and down regulation of *E2F*. It is evident that the low levels of *cyclin E *could hypo-phosporylate Rb and fail to release *E2F *rendering them in their repressive forms resulting in S phase arrest. We have also observed an up regulation of *Zak *proteins which are members of Mitogen activated protein kinase pathway (*MAPKKK*) that triggers cell cycle arrest and check point regulation following stress response. Zak has been associated with down regulation of cyclin E and S/G2/M arrest [30]. Therefore, we suggest that events like Zak down-regulating cyclin E which represses *E2F *and decrease cyclin B levels by Gadd45 resulted in the observed cell cycle arrest. Reports have already emphasized role of p53 in regulating PCNA and p21 genes [31]. Hence, down regulation of p53 is believed to be the reason for low levels of p21 and PCNA which might play a role in determining the cellular fate. Along with the down regulation of p53 expression we also observed phosphorylation of residual p53 in treated cells probably facilitating p53 mediated effects.

Expression patterns of genes involved in DNA damage response pathway suggest that cells treated with Ag-np suffered impaired DNA repair mechanisms in normal cells and initiation of DNA repair response in cancer cells. This is evidenced by the increase in ATM and ATR levels indicating double strand DNA breaks where they act as sensors activating downstream targets for DNA repair [32]. Normal cells showed down-regulation of genes involved in base excision repair (*Mbd4, Apex1, OGG1 and Mutyh*), mismatch repair pathways (*Mutyh, Abl1, Pms1 and MSH2*) and double strand break repair pathway (ATM). These molecular results further confirm existing genotoxicity data, where DNA damage and chromosomal aberrations were observed to be higher in Ag-np treated U251 cells than IMR-90 [26]. Up regulation of Abl and Brca1 genes specifically in cancer cells in contrast to that of the normal cells is a significant observation. DNA repair pathway responsible for DSBs repair such as homologous recombination (HR) is likely to be activated with up-regulation of Brca1. Non-homologous end joining (NHEJ), another major DSBs repair mechanism does not play important role in DSBs repair in our model as up-regulation of Abl will down-modulate NHEJ pathway [33,34]. PCNA is a co factor for DNA polymerase and an essential player in DNA synthesis and DNA repair [35]. Down regulation of PCNA following Ag-np exposure could have indicated that repair mechanisms such as base excision repair, nucleotide excision repair and mismatch repair are not essential in this study as mostly DSBs were induced.

Cell adhesion and extracellular matrix genes were not highly affected by nanoparticles exposure. However, up regulations of alpha integrins were observed in both cell lines. Integrins play vital role in signal transduction and interactions of cells with extracellular matrix. Up regulation of integrin operates protective mechanisms where cytoskeletal and ECM are challenged by extraneous factors [36]. Interestingly, an up regulation of tumour growth factor or *Tgfbi *was observed in treated cancer cells. However, no *TGF *over expression was detected in normal cells.

Signalling pathway analysis identified up regulation of *jun *in both cell lines which can act through JNK pathways regulating cell proliferation or cytokine production. Involvement of JNK pathway has been reported in silver nanoparticle mediated toxicity studies [37]. Our experiments unveiled involvement of JNK pathway as over expression of transcription factor *Jun*. NFκB is a major transcription factors which is a key regulator of immune survival and anti-apoptotic response. *NFκB1 *and *NFκBIA *are seen over expressed in cancer cells. A significant decrease in the expression of NFκB was observed in normal cells following Ag-np exposure. On the other hand an increase in the expression of *MAPK *was observed in normal cells. *Jun *up regulation was accompanied by up regulation of pro-inflammatory cytokine *IL-8*, granulocyte macrophage colony stimulating factor (*GM-CSF*) in normal cells. Similar observations were reported in carbon nanoparticle toxicity where IL-8 production was observed to be mediated by MAPkinase pathway [38]. Another regulator of signalling cascades which was differential expressed is Gadd 45. It is activated by cytokines and signal transduction pathways like MAPkinase pathway [39]. Once activated, Gadd 45 can trigger MAPKinase pathway as well.

Up regulation of *Bax *in cancer cells following Ag-np exposure, suggested induction of apoptotic stimuli through mitochondrial pathway. None of the apoptosis related genes were up regulated in normal cells. Down regulation of survivin, an apoptosis inhibitor protein *(IAP) *that blocks caspases renders it susceptible to apoptosis. Survivin is over expressed in cancer which increases resistance to apoptosis [40]. The differential gene expression profile observed following Ag-np exposure exhibits selective induction of pathways in normal and cancer cells which will determine the nature of response to nanoparticle exposure.

### Release of pro-inflammatory cytokines from silver nanoparticles treated fibroblasts

The current investigation shows the ability of silver nanoparticles to trigger an inflammatory response based on induction of various pro-inflammatory cytokines and chemokines. A threefold increase in IL-6 levels was observed subsequent to exposure with silver nanoparticles. A similar increase in IL-6 levels following silver nanoparticle exposure recently reported corroborates with our observation [41]. IL-6, produced by a variety of cells including fibroblasts is a pleiotropic cytokine involved in the amplification of inflammatory responses [42,43]. Rapid increase in IL-6 levels is observed during acute inflammatory responses due to injury, infection and stress [42,43]. It induces systemic effects affecting various organs and has been associated with a plethora of clinical conditions such as inflammatory lung disorders [44] and rheumatoid arthritis [45]. We have observed an eight fold increase in the levels of interleukin 8, which is also produced by a variety of cells, including fibroblasts [46]. Inappropriate increase in the levels of IL-8 has been associated with inflammatory disorders as well [47]. Intercellular adhesion molecule -1 (ICAM-1), MIP-1, MCP-1 (Monocyte chemoattractant protein - 1) and GRO-alpha known to be up regulated in inflammatory conditions was also seen following our silver nanoparticles exposure. These modulate chemotaxis, degranulation, phagocytosis and signalling mediator synthesis in cells [48,49]. Dysregulation of MIP-1, MCP-1 and GRO-alpha have been associated with a variety of inflammatory disorders [50-52]. We also observed an increase in the levels of GM-CSF and IFNγ. GM-CSF is involved in chemotaxis, phagocytosis and antigen presentation [53,54]. Increased levels have been associated with rheumatoid arthritis, chronic obstructive pulmonary disease and asthma [54]. IFNγ responds against intracellular pathogens and tumour control. Abnormal expression and protein levels of IFNγ have been associated with autoimmune and inflammatory conditions [55].

There are reports regarding the anti-inflammatory effects of silver nanoparticles [15,56,57]. However, the nature of the study (objectives, experimental conditions and endpoints) should be taken into consideration. Most of the studies were conducted on *in vivo *models (disease or wound healing models) with pre-existing injuries and utilized different combinations of silver nanoparticles coated/impregnated wound dressings. The objective of these studies was to analyze the additive therapeutic effects silver nanoparticles can have on the diseased or injured tissue. Those studies which reported similar findings using in-vitro studies evaluated the levels of these cytokines following an addition of a stimulus like LPS while pre or co-incubating with the silver nanoparticles. Our study was designed to evaluate the effect of these nanoparticles on normal unstimulated cells. Moreover, it should be noted that the source of the nanoparticles, concentration, size, technique and stabilizing agents involved in the preparation will all influence the response. To summarize, our study clearly shows an increase in the pro-inflammatory cytokines specific for human fibroblasts. This can be a unique response signature in these cells which should be taken into consideration. Though our study suggests an inflammatory response following Ag-np exposure, we cannot rule out the possibility of modifiers such as cell type used and nanoparticle used in the study.

Ag-np coated medical devices and wound dressings are already in the market. Human beings are constantly exposed to such commercial products the biological properties of which are still elusive. This study sheds light in to possible side effects of silver nanoparticles used in cosmetics, household products and in wound dressings. It is tempting to speculate that Ag-np impregnated wound dressings, catheters and other medical devices could induce similar response which may have detrimental effects *in vivo *as evidenced by the induction of DNA damage and inflammatory response by Ag-np in our study.

## Conclusion

In summary, the genomics data suggests involvement of genotoxic stress and subsequent down regulation of DNA repair pathways in IMR-90 cells and U251 cells. Improper DNA repair could be a reason for accumulating cells at S/G_2_/M phases of the cell cycle. Ag-np is able to down regulate many proteins involved in cell cycle progression and DNA repair by preventing their degradation possibly by surface adsorption. Ag-np can activate a variety of pathways including MAPK and NFκB pathways resulting in transcription of many genes involved in proliferation and inflammatory response. Our findings also suggest that only a small population of cells are undergoing apoptosis while majority are alive and were undergoing proliferation arrest. Our data reveal that Ag-np toxicity could trigger a detrimental inflammatory response. A more in-depth and multi parametric studies are recommended to elucidate the diverse role and effects of nanoparticles on different types of human cells including cancer cells. Such studies will further facilitate accurate toxicological profiling of nanoparticles and their potential therapeutic use in the management of different pathological conditions in humans.

## Methods

Nanoparticles employed in this study (6-20 nm) were synthesised and characterised by us as described in our previous reports [26,58]. Extensive characterisations of the particles have been done through electron microscopy, dynamic light scattering and UV-visible spectroscopy.

### Cell culture and nanoparticle exposure

Normal human lung fibroblasts (IMR-90) were purchased from Coriell cell repositories, USA. Cells were maintained in Minimum Essential Medium (MEM, Invitrogen, NY USA) supplemented with 15% foetal bovine serum (Hyclone, USA), 2% essential amino acids and 1% each of non-essential amino acid, vitamins and penicillin-streptomycin (GIBCO, Invitrogen, USA). The cells (passage 14 ± 2) were grown at 37°C in the presence of 5% CO_2 _at log phase. Human glioblastoma cells were received from Dr. Masao Suzuki, National Institute of Radiological Sciences, Chiba, Japan and were maintained in Dulbecco's modified Eagles medium (DMEM, Sigma-Aldrich, MO, USA) supplemented with 10% foetal bovine serum (FBS, GIBCO, Invitrogen, USA) and 1% penicillin streptomycin (Gibco, Invitrogen, NY, USA). Stock solutions were prepared in ultrapure water by dissolving 5 mg of silver nanoparticles in 1 mL of ultra pure water. A uniform suspension was prepared by sonication.

### Isothermal titration calorimetry

Isothermal titration calorimetry (ITC) computes the kinetics of molecular interactions based on the thermodynamics in the reaction. The interaction between the ligand and the binding partners is measured through subtle heat changes occurring in the reaction chamber. The system consists of an injector, sample chamber and a reference cell. The ligand is injected at regular intervals into the chamber containing the second molecule. The sample chamber and the reference cells are kept at constant temperature. The binding between the ligand and the partner can either release or absorb heat. Endothermic (absorbs heat) reactions will prompt the system to provide heat in order to maintain the standard temperature in relation to the reference cell, whereas an exothermic reaction initiate absorption of excess heat by the system to stabilize the temperature. Heat changes per injections will be used to assess the interaction and calculate the binding affinity between the molecules. ITC experiments were carried out using a VP-ITC titration calorimetric system (Microcal Inc., MA, USA) operated at 25°C. Each microcalorimetric titration experiment consisted of 60 successive injections of a constant volume (5 μL/injection) of cytosolic protein (300 μg) in water to the reaction cell containing (150 μg in 1.8 mL) Ag-np. The binding of starch to protein solutions in the absence of Ag-np and binding of starch capped Ag-np with genomic DNA was determined, using the same number of injections and concentration of proteins/DNA as in the titration experiments. Data analysis was carried out with Origin 7.0 software supplied with the instrument.

### Messenger RNA isolation and array hybridisation

Cells were seeded at a density of 2 × 10^6 ^cells/T75 flasks and treated with 400 μg/mL of Ag-np for 48 hours. Cells were harvested in cold 1 × phosphate buffered saline (PBS, 1^st ^Base, Singapore) and mRNA was isolated using Qiagen RNeasy mini kit (Qiagen, Hilden, Germany), following manufacturer's instruction. The concentration and integrity of mRNA was tested using Nanodrop spectrophotometer (Thermo Fischer Scientific, USA). The RNA from untreated and nanoparticle treated cells were used for hybridisation. The arrays (Oligo GEArray; SuperArray Bioscience Corporation, MD, USA) employed were human cell cycle oligo GEArray (OHS-020), human DNA damage signalling pathway oligo GEArray (OHS-029), human signal transduction pathway finder oligo GEArray (OHS-014) and human extracellular matrix and adhesion molecules oligo GEArray (OHS-013). Probing was done as per manufacturer's instructions. Briefly, complementary RNA (cRNA) was synthesised using TrueLabeling-AMP Linear RNA Amplification Kit (SuperArray Bioscience Corporation, Frederick, MD) as per manufacturer's instructions and biotinylated using Biotin-16-uridine-5'-triphosphate (Roche, Germany). The biotinylated cRNA (3 mg) was used for overnight hybridisation at 60°C. The membrane was treated with alkaline phosphatase conjugated to streptavidin. Treated membranes were exposed to chemiluminescence reagent and image was captured on X- ray films. Image analysis was performed using GEArray expression analysis suite (SuperArray Bioscience Corporation, MD, USA). Normalisation was carried out using *Rps27a, Gapdh, B2m, Hsp90ab1 *and *Beta actin*. All values were normalised against housekeeping genes. Fold change is calculated as treated/control - 1. Basal levels were normalised to 0 instead of 1.

### Real-time Reverse Transcriptase Polymerase Chain Reaction (RT-PCR)

RT-PCR was used to validate the results from array. RT-PCR was done using LightCycler^® ^RNA Amplification Kit SYBR Green I (Roche, Germany). Probes were designed using primer design utility. The details of genes employed and annealing temperature (T_A_) are included in Table 6. DNA strands were amplified by denaturation at 95°C for 30 seconds followed by annealing of primers at specific temperatures for 15 seconds. Extension of the strands was done for 1 min at 72°C. The experiments were conducted three times independently.

**Table 6:** Primer sequences and conditions employed in RT-PCR

Gene	Primer sequence	T_A _(°C)
p53	5'GGC CCA CTT CAC CGT ACT AA3' 5'GTG GTT TCA AGG CCA GAT GT3'	60
Mitogen activated protein kinase (MAPK1)	5'CCA CCC ATA TCT GGA GCA GT3' 5'CAG TCC TCT GAG CCC TTG TC 3'	60
Interleukin-8 (IL-8)	5'ATG ACT TCC AAG CTG GCC GTG GCT 3' 5'TCT CAG CCC TCT TCA AAA ACT TCT C 3'	61
Nuclear factor beta (NFκB)	5' AGT TGA GGG GAC TTT CCC AGG 3' 5' GCC TGG GAA AGT CCC CTC AAC T 3'	61
18s Ribosomal subunit	5' GTA ACC CGT TGA ACC CCA TT-3' 5' CCA TCC AAT CGG TAG TAG CG 3'	60

### Western blotting

Cells density and exposure were maintained similar to array experiments. Cells were scraped and washed with cold 1 × PBS. Protein was isolated by lysing the cells on ice for 20 min with radio immuno precipitation buffer (RIPA, 100 μL). The protein was isolated by centrifugation at 12,000 rpm for 1 hr at 4°C and quantified using Bradford assay kit with bovine serum albumin (BSA) standards (Biorad laboratories, CA, USA). Proteins (40 μg) were separated in 10% poly-acrylamide gel and blotted on to nitrocellulose membrane (Hybond ECL, Amersham biosciences, UK), blocked in 5% non-fat milk and incubated with primary antibodies (monoclonal anti mouse antibodies) against PCNA (dilution 1:800), cyclin B1 (1:1000), p21 (1:500), p53 (1:2000) phosphorylated p53 (phospho p53, cell signalling technologies Inc, Danvers, MA, USA 1:1000), PARP (1:1000) survivin (1:500), caspase 3 (1:500), and beta actin (Chemicon international, Temecula, CA, USA 1:5000). All antibodies were purchased from Santa Cruz biotechnologies Inc (Santa Cruz, CA, USA) unless stated otherwise. Goat anti-mouse antibody tagged with horseradish peroxidase (Immuno pure, Pierce biotechnologies, Rockford, IL, USA, 1:5000) was used as secondary antibodies. Protein bands were visualized by chemiluminescence (ECL, Amersham biosciences, UK) and exposed to X-ray films (Pierce biotechnologies, Rockford, IL, USA). The intensity of bands was analysed using Kodak molecular imaging software (Kodak, USA) and normalised against actin. The normalised values were used for comparison with array data. Additional experiments were done using selected proteins (PCNA, cyclin B, p53, p21, caspase 3, PARP) for Ag-np concentrations 50 and 200 μg/mL, to explore dose dependant effects.

### Immunofluorescence staining for γH2AX

Phosphorylation of histone H2AX occurs following a double strand DNA break, where ATM/ATR is activated. The phosphorylated protein binds to the DNA, at the damage site, aiding the repair process, which can be used as a biomarker for detecting ds DNA damage. The cells were plated on cover slips (0.4 million cells/well) in 6-well plates and treated with the nanoparticles (25, 50 and 100 μg/mL of Ag-np-3; 80 and 160 μg/mL of Pt-np) for 2 hrs. Cells exposed to 10 μM H_2_O_2 _were used as positive controls. Following incubations, the cover slips were washed in PBS twice and fixed in 4% formaldehyde for 15 min. After washing away excess formaldehyde, the cells were permeabilised in 0.2% triton × solution, blocked in 5% BSA (1 hr) and incubated in primary Anti-phospho histone γH2AX mouse monoclonal antibody (Ser 139, JBW 103, Upstate biotechnology Inc., USA) for 1 hr. The cover slips were washed thrice in PBS to remove excess of primary antibody and stained with secondary antibody (anti-mouse antibody conjugated to FITC, eBiosciences, USA) for 1 hr. After washing away the excess of secondary antibodies, the cover slips were mounted in DAPI-vectashield. The images were captured under the DAPI-FITC filter of Zeiss Axioplan microscope. The experiment was repeated three times. Fifty DAPI-stained nucleus per sample were captured randomly with Zeiss Axioplan 2 imaging fluorescence microscope equipped with appropriate filters. Number of foci on each cell was recorded.

### Cytokine detection assay

The effect of Ag-np on the levels of cytokines and chemokines of human lung fibroblasts were determined by collecting the supernatants from both the untreated and silver nanoparticles treated cultures were collected and stored in -80°C until used. The cytokines were quantified using Bio-Plex™ Cytokine assay (Bio-Rad Laboratories, CA, USA). Premixed multiplex beads of the Bio-Plex human cytokine panel, which included 17 cytokines of choice (IL-1β, IL-2, IL-4, IL-6, IL-7, IL-8, IL-16, IL-17, TNFα, IFNγ, MCP-1 (CCL2), MIP-1α (CCL3), MIP-1β, RANTES, ICAM-1, GM-CSF, GROα) were used and the assay was performed thrice by strictly adhering to the manufacturer's instructions. The data were analysed using the Bio-Plex Manager 4.0 software (Bio-Rad Laboratories, CA, USA).

## Competing interests

The authors declare that they have no competing interests.

## Authors' contributions

AR, MPH and SV designed the experiments. AR performed nanoparticle synthesis, ITC, western blots and super array and conducted the experiments. GB analyzed ITC data. SS conducted and analyzed cytokine profiling. AR and HKL wrote and edited the manuscript. All the authors approved the final version of the manuscript.
